# Reliability of Agreement between Insulin, Clonidine, and Glucagon Stimulation Tests for the Diagnosis of Growth Hormone Deficiency in Children: A Retrospective Cohort Study

**DOI:** 10.3390/children10081381

**Published:** 2023-08-14

**Authors:** Rana Al Balwi, Mohammad Al-Qahtani, Amani K. Alrowished, Hwazen A. Shash, Reem Alatrash, Alanoud Alhareth, Roba Aldossary, Maha Alahmari, Yara Hejazi, Alia Alammari, Sarah AlShawaf, Rawan Al Balwi, Suzan AlKhater

**Affiliations:** 1 Division of Pediatric Endocrinology, Department of Pediatrics, King Fahad Hospital of the University in AL Khobar, Imam Abdulrahman Bin Faisal University, Dammam 34212, Saudi Arabia; 2 College of Medicine, Imam Abdulrahman Bin Faisal University, Dammam 34212, Saudi Arabia; 3 Department of Emergency Medicine, King Fahad Hospital, Ministry of Health, Jeddah 11176, Saudi Arabia

**Keywords:** pituitary dwarfism, inter-observer reliability, glucagon, clonidine, insulin, growth hormone, somatotropin

## Abstract

Growth hormone (GH) deficiency (GHD) is a rare disorder. The diagnosis of GHD requires a combination of two provocative GH tests. This study aimed to find agreement between commonly used medications to determine which combined tests have high reliability of agreement. This retrospective cohort included 201 children who underwent GH provocation testing from January 2012 to December 2022. The insulin tolerance test (ITT) with the clonidine stimulation test (CST) or glucagon stimulation test (GST) with the CST were performed. We calculated Cohen’s kappa to determine the agreement between the test medications by considering the post-stimulation peak GH level with a cut-off value of 10 ng/mL as the primary outcome. A total of 151 patients underwent the two provocative tests and were included in the analysis. Of these patients, 119 underwent the ITT and CST and 54 (45.3%) were diagnosed with GHD. However, 32 patients underwent the GST and CST and 18 (56.2%) were diagnosed with GHD. The kappa value for ITT and CST was 0.258 (25.8%), indicating fair agreement between clonidine and insulin (*p* = 0.005). However, the kappa value for CST and GST was 0.178 (17.8%), representing slight agreement. The correlation coefficient revealed a very strong relationship between ITT and CST. Clonidine has fair agreement and a very strong correlation coefficient with ITT when used to diagnose GHD in children. Among the commonly used pharmacological tests for GH provocation in our unit, the CST was considered the best pharmacological test in terms of safety and reduced parental anxiety.

## 1. Introduction

Growth hormone (GH) deficiency (GHD) is a rare disorder with a prevalence of approximately 1 in 4000 during childhood [1]. The GHD diagnosis depends on the measurement of the peak GH level secreted by the pituitary gland. GHD is primarily a clinical diagnosis that is confirmed by provocative GH tests [2]. A conventional standard has proposed that the diagnosis of GHD requires inadequate peak GH concentrations according to two provocative GH tests (which can be performed sequentially on the same day) because of the high frequency of false “failure” results of any provocative test.

Notably, the generally accepted diagnostic GH threshold is 10 mcg/L [1]. However, combining the two most commonly used provocative GH tests results in poor specificity. However, a new cut-off limit of 7.09 mcg/L using the iSYS isotope dilution mass spectrometry assay has been accepted as an international standard for diagnosing GHD in children [3].

It has been proven that the GH response and test specificity decrease with the body mass index (BMI), even for those with a BMI within the normal range [4], and that children with obesity have particularly low GH concentrations. However, the lack of a gold standard for diagnosing childhood-onset GHD compels clinicians to determine the diagnosis based on a combination of factors, including physical appearance, short stature, presence of other hormone deficiencies, and low height velocity, among others [2]. Medical imaging abnormalities of the hypothalamic–pituitary area [5], low insulin-like growth factor-1 (IGF-1) levels, IGF-binding protein-3 levels [6], and GH provocative test peaks below an arbitrary cut-off (10 mcg/L) are also used as diagnostic tools. However, a definitive diagnosis of partial or mild GHD remains difficult and uncertain [7]. Many difficulties with provocative GH tests have been highlighted, including the limited validity of indicators of the physiological GH secretory capacity and poor reproducibility of the test method [8,9,10], which is attributed to the significant normal intra-individual and inter-individual variations in responses [11]. 

Despite knowing that hypothalamic regulation of pituitary somatotrophs is controlled by the stimulatory effects of GH-releasing hormone (GHRH) and ghrelin as well as the inhibitory effects of somatostatin, the sources of these variations have not been well-documented. Amino acids stimulate GH, whereas IGF-1 mediates GH action and inhibits GH secretion in a negative feedback loop [12].

Different medications have been used for GH stimulation tests. The most commonly used are insulin, clonidine, and glucagon. Compared to glucagon, clonidine has a significantly lower error rate when used for GH stimulation tests; therefore, clonidine was the focus of a retrospective, single-center, observational study involving 512 children [13]. Insulin is not preferable for use in children because of its major adverse effect (hypoglycemia). However, two studies evaluated peak GH level variability using insulin, and both concluded that the time after insulin injection determines the severity of hypoglycemia, with durations less than 90 minutes indicating safety and cost-effectiveness [14,15].

In clinical settings, it is common to encounter patients who are anxious about sequentially undergoing two tests, those who do not attend the appointment on the test day, those who ask to be discharged without undergoing the second test, and those who refuse to continue testing after developing symptoms such as headache, nausea, and drowsiness. Therefore, it is important to know which combined tests can be considered reliable for use on the same day. This study aimed to determine the agreement between the stimulation tests commonly used in sequence on the same day at our unit.

## 2. Materials and Methods

### 2.1. Study Design

This study included 201 retrospective cohort cases of patients between 4 and 14 years of age between January 2012 and December 2022. The institutional review board of the center provided ethical approval for data collection. The patients were initially evaluated in the clinic for short stature, and those with suspected GHD were referred for the GH provocation test. The criteria for suspected GHD were based on consensus guidelines for diagnosing GHD in childhood that were published in 2000 [16,17] and recommend investigation of the following presentations: (1) severe short stature, defined as height >3 standard deviations (SDs) below the mean; (2) height velocity >1.5 SDs below the mid-parental height; (3) height velocity >2 SDs below the mean and height velocity >1 SD below the mean sustained over 1 year for chronological age or a decrease in the height SD >0.5 sustained over 1 year for children older than 2 years of age; (4) in the absence of short stature, a height velocity >2 SDs below the mean sustained over 1 year or more than −1.5 SDs sustained over 2 years, which may occur with GHD presenting during infancy or organically acquired GHD; (5) signs indicative of intracranial lesions; and (6) signs of multiple pituitary hormone deficiencies. 

The included patients underwent GH provocation testing using two medications and two separate tests in 1 day. The stimulation tests were the insulin tolerance test (ITT) with the clonidine stimulation test (CST) and the glucagon stimulation test (GST) with the CST, which followed a standard protocol. With the ITT, intravenous insulin was administered (0.05–0.1 U/kg) following an overnight fast, and blood samples were obtained at 0, 15, 30, 60, and 90 min to determine GH, glucose, and cortisol after the nadir glucose level. With the CST, clonidine was administered orally at 0.15 mg/m^2^ body surface area between 8:00 am and 9:00 am after fasting overnight, and blood samples were collected at 0, 30, 60, 90, and 120 min to determine GH levels. The GST was performed after an overnight fast by subcutaneously injecting 1 mg glucagon (Novo Nordisk, Bagsværd, Denmark). Blood samples for GH measurements were obtained before the glucagon injection (baseline value) and after 90, 120, 150, 180, 210, and 240 min. 

The results were interpreted by a pediatric endocrinologist in the clinic using 10 ng/mL as a cut-off value. GH was measured in the laboratory using the chemiluminescent microparticle immunoassay test with the lowest detectable value of 0.05 ng/mL (1 ng/mL = 1 mcg/L). With all tests, glucose monitoring and vital sign recordings were performed every 15 to 30 min under the close supervision of a nurse and endocrinologist. Diagnostic labels of “positive” and “negative” were used to define the low post-stimulated peak GH level (≤10 ng/mL) and high post-stimulated peak GH level (≥10 ng/mL), respectively. These diagnostic labels were used to create the experimental study design and ensure the correct distribution across the diagnostic groups. 

The data collection sheet used to record patient characteristics included age, sex, height (cm), weight (kg), Tanner stage of puberty, BMI SD score, mid-parental height, bone age, baseline IGF-1 level, height velocity over 12 months (growth velocity cm/12 months), thyroid function, celiac screening, adrenocorticotropic hormone level, cortisol level, luteinizing hormone level, follicle-stimulating hormone level, prolactin level, underlying diagnosis, medication used for stimulation, documentation of any abnormal vital sign records or any adverse events associated with any medications used, and causes of test termination. The primary outcome was the peak post-stimulation GH level.

### 2.2. Statistical Analysis

Statistical inter-rater reliability was computed to evaluate agreement across the three medications (raters), insulin, clonidine, and glucagon, using Cohen’s kappa for the post-stimulated peak GH level of the positive and negative test results [18]. The annotated data were then categorized (positive or negative), and agreement between raters was evaluated [19]. Cohen’s kappa was calculated using IBM SPSS statistics version 29.0.0.0 (214) (IBM Corp., Armonk, NY, USA) [20] for all rater pairs (insulin with clonidine and glucagon with clonidine), providing an overall metric of agreement. Cohen’s kappa coefficient ranges between 0 and 1, and the scales of interpretation are as follows [21]: slight agreement, 0 to 0.2; fair agreement, 0.21 to 0.4; moderate agreement, 0.41 to 0.6; substantial agreement, 0.61 to 0.8; and perfect agreement, 0.81 to 1. This study reported the percentage agreement and kappa, as suggested by Myburgh et al. [22], because they each have advantages and limitations. Furthermore, the correlation coefficient was computed for the data using Spearman’s rank correlation coefficient using SPSS to assess the strength of the relationship between the variables (ITT with CST and GST with CST) and display it on a scatter plot.

Both correlation coefficients are scaled because they range from −1 to +1 and indicate whether there is a monotonic association or constantly increasing or decreasing curve based on the Dancey and Reidy scale created in 2004 [23].

## 3. Results

**Figure 1:**
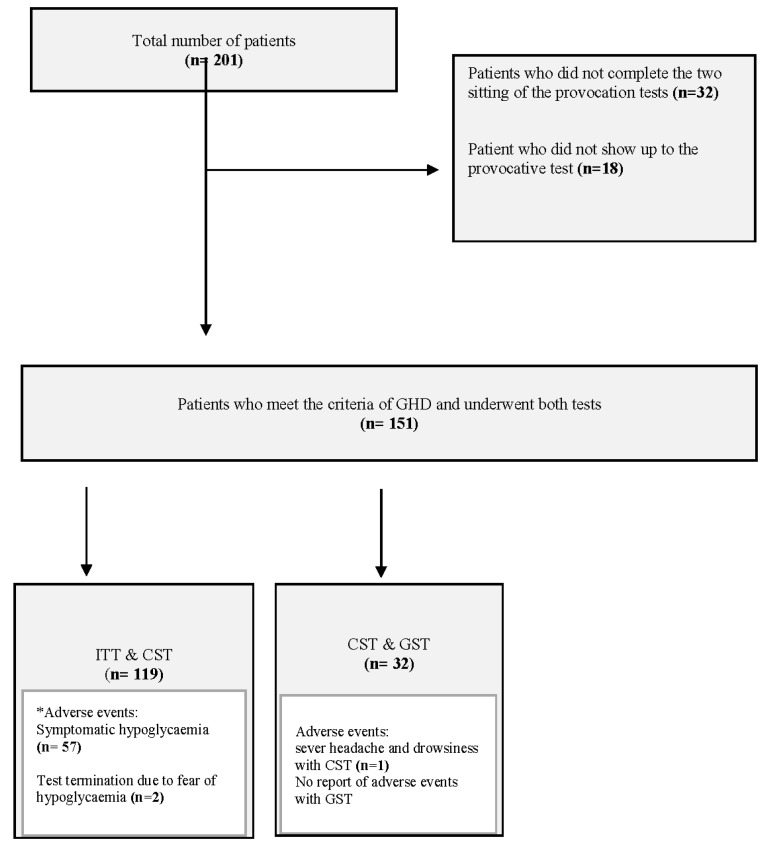
Flowchart identifying the patients included in the study. CST = clonidine stimulation test; GST = glucagon stimulation test; ITT = insulin tolerance test. * Convulsion and loss of consciousness were not reported with the ITT.

A total of 201 children were included in this study between January 2012 and December 2022. Table 1 presents the patient characteristics. However, 50 patients were excluded from the study; of these patients, 18 did not attend the provocation test appointment and missed their follow-up appointment, and 19 did not complete two provocation tests. Furthermore, 13 of these 19 patients declined to undergo the second test because of the side effects, which included drowsiness that occurs with hypotension with the CST (four patients) and the development of hypoglycemia with the ITT that resulted in symptoms comprising tremors and headache (nine patients, including three for whom the ITT was terminated upon the request of the parents). Abnormal movements and loss of consciousness with hypoglycemia were not reported, but the remaining six patients terminated the test after the first episode of hypoglycemia. Additionally, 13 patients refused a prolonged stay in the day-care unit. Finally, 151 patients who underwent two provocation tests were included in the analysis (Figure 1).

Among these 151 patients, 119 underwent the ITT and CST, and 54 (45.3%) were diagnosed with GHD. Adverse events in 57 patients who successfully completed the ITT were reported, including headache and drowsiness; however, severe symptoms such as convulsion or loss of consciousness were not reported. At the request of the parents, the ITT was terminated for two patients after they developed symptomatic hypoglycemia (headache and drowsiness). However, 32 patients completed the CST and GST, and 18 (56.2%) were diagnosed with GHD; one case of hypotension (severe headache and drowsiness) during the CST was reported. No adverse effects during the GST were reported (Figure 1).

### 3.1. Inter-Rater Reliability 

Cohen’s kappa was calculated for the two groups to identify their agreement when used to diagnose GHD (the ITT with CST and the GST with CST). Table 2 shows the summary of the results. The kappa value was 0.258 (25.8%), indicating fair agreement between clonidine and insulin; this result was statistically significant (*p* = 0.005). As illustrated in Table 3, the kappa value was 0.178 (17.8%), representing slight agreement between glucagon and clonidine; this result was not significant (*p* = 0.314). 

### 3.2. Correlation Coefficient

**Figure 2:**
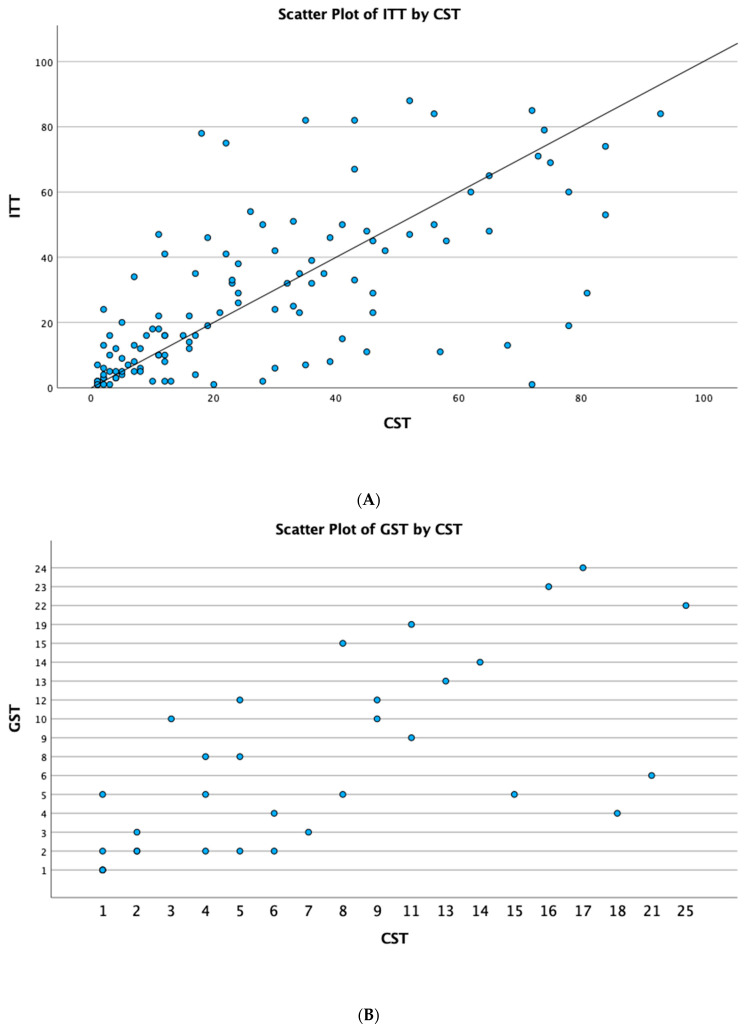
(**A**) Scatter plot of the ITT and CST. (**B**) Scatter plot of the GST and CST.

Spearman’s rank correlation coefficient calculated the values of the ITT and CST. Table 4 (A) reveals a correlation coefficient of 0.710, which indicates a very strong relationship between the ITT and CST (monotonic correlation). Moreover, Table 4 (B) reveals a very strong relationship between the values of the GST and CST, with a correlation coefficient of 0.702. Figure 2 displays the scattered plots.

## 4. Discussion

This study confirmed fair agreement between the ITT and CST for diagnosing GHD in children, with a statistically significant result. In contrast, based on Cohen’s kappa value, this study objectively validated slight agreement between the GST and CST for diagnosing GHD in children. The peak GH level used as the variable during this study had only two possible states (positive or negative), and these states were sharply differentiated. Therefore, reliability was likely to be high. Furthermore, kappa values may have wide confidence intervals that include good to poor agreement [24]. Therefore, the correlation coefficient was calculated to determine the association.

This low level of agreement between the CST and GST could explain the discrepancy in the post-stimulated peak GH levels (between the CST and GST) that is occasionally observed during the interpretation of the result of the same patient because the peak GH level of the CST corresponds with the clinical features of GHD; however, the GST indicates a high post-stimulated peak level considering that the GST and CST were performed separately during 1 day. Therefore, it is presumed that the GST is less reliable than the ITT and CST, and a lower cut-off value (less than 7 ng/mL) could be proposed for GST during future research. Furthermore, we found it reasonable to assess the agreement of the CST in relation to the ITT because the ITT has been traditionally accepted as the gold standard test for assessing adult GHD [25] because of its sensitivity and reproducibility [26], and because it enables the simultaneous assessment of the entire hypothalamic–pituitary–adrenal axis [27]. 

This study found that the occurrence of parental anxiety after the ITT precluded the performance of the second test. Hypoglycemia was the major adverse event that triggered fear among the parents of the patients who underwent the ITT with the CST. However, the side effects of the CST (headache and drowsiness) did not generate the same level of parental anxiety or lead to test termination. 

A systematic survey [28] that was conducted at more than 18 endocrine units to assess the controversy associated with performing the ITT for children revealed that a sense of fear regarding extreme hypoglycemia appeared to be the dominant reason for not performing the ITT at these units, followed by the event of convulsions. Kaplan et al. [29] published their findings regarding insulin-induced hypoglycemia as a provocation test for 134 children with short stature and concluded that it was valuable for distinguishing children with GHD when it occurs as an isolated deficiency from children with other forms of growth restriction; however, it did not provide insight regarding other forms of growth restriction. Investigators from Glasgow reviewed their experience of performing 550 ITTs over the course of 10 years (1989–1999) [30] and reported that no severe adverse events occurred during that period, thus concluding that the ITT is a safe and reliable test for children when a strict procedure is followed [17].

Furthermore, regarding the CST for children, clonidine has been associated with hypoglycemia; and the mechanism of action is unclear [31]. However, during a study of the performance of 225 CSTs, the side effects of clonidine administration were observed in 23% of the patients [32]; this result was consistent with that reported by another study [33]. Only somnolence and mild hypotension were observed and did not require oral hydration or saline infusion, as reported by several studies [33,34]. Regarding convenience and cost-effectiveness, another study assessed whether minimizing the sampling points could reduce costs while maintaining the efficacy and sensitivity of the test and concluded that a sample obtained 60 min after clonidine stimulation was the best single sample for ruling out GHD, with 79.5% specificity [35]. However, Gillis et al. reported that the peak indicating GH sufficiency (85.15%) during the CST tended to occur more frequently at typical times (60 min and 90 min) than those indicating GHD (68%) [36]. 

Lim et al. reported a high rate of hypoglycemia during the GST, particularly for children younger than 8 years of age [37]. A total of 27 of 80 children (33.8%) developed hypoglycemia with a blood glucose nadir of 52.2 mg/dL (2.9 mmol/L), and seven children successfully received oral hypoglycemia treatment. Moreover, Hanukoglu et al. [28] reported that the glucagon test was interrupted occasionally because of unpleasant side effects (weakness, nausea, and vomiting) and rarely because of severe hypoglycemia, rather than because of convulsions. Many endocrinologists consider the GST to be a valuable alternative to the ITT, thus facilitating the simultaneous assessment of corticotroph and somatotroph functions in children [38]. However, because glucagon is a potent insulin secretagogue, it is crucial to consider the previously reported studies that indicated that glucagon may result in rebound hypoglycemia [39]; furthermore, it is important to consider that the adrenal response was age-dependent and sex-dependent during the GST, associated with a high false-positive rate (23.7%), and resulted in the overdiagnosis of adrenal insufficiency in 190 children [40]. 

Based on the results of this study, and considering the previously reported findings, we concluded that the CST could be considered a relatively safe test for diagnosing GHD in children with close monitoring of vital signs. Moreover, based on the observations in the clinical setting regarding the reliability and accuracy of the GST, it is recommended that the GST accuracy for diagnosing GHD should be assessed by a large-scale study with a lower cut-off value of the post-stimulated GH level (7 ng/mL). Additionally, age is likely an important factor that determines how people respond to various GH-stimulating agents. Glucagon is a well-established agent for adults, but it may be less reliable when used for children [41]. 

Moreover, it is important to consider the possibility that the most common reason for test results that indicate GHD is the physiological absolute, followed by the relative refractory period after a natural peak of spontaneous GH secretion (false-positive results).

### Strength and Limitations 

This study focused on the reliability and agreement of GH provocative tests among a large number of patients and objectively confirmed the reliability of agreement (inter-rater reliability) between the ITT with CST and the GST with CST. Additionally, we were able to assess the Spearman’s rank coefficient correlation to determine robust agreement, especially with glucagon, which was found to have a response higher than that of other medications. We reviewed the literature to ensure the safety of the CST and GST for children and correlate the observations of adverse events and parental anxiety in the clinical setting with those reported. 

This study had some limitations. First, blood glucose monitoring in the day-care unit did not depend on real-time continuous glucose monitoring. Second, the ITT and CST were performed in 1 day, and the ITT could not be the diagnostic gold standard for patients who subsequently underwent the CST. Finally, we did not investigate confounders, including sex variability, puberty level, and BMI percentage, during the analysis. In conclusion, this study indicated fair inter-rater reliability and agreement of the CST with the ITT; therefore, in terms of safety, the CST could be considered a perfect replacement for the ITT for children. 

## Figures and Tables

**Table 1:** Patients’ characteristics.

Parameters	Mean ± SD
Age (years)	10.03 ± 2.88
Height (below the age and sex standard means)	−2.3 ± 1.2
BMI (kg/m^2^)	15.95 ± 4.053
Tanner stage of puberty	2 ± 1.2
Growth velocity (cm/12 months)	3.074 ± 2.093
Bone age (years)	8.34 ± 4.51
Peak GH (ng/mL)	
With insulin	5.79 ± 3.01
With clonidine	5.85 ± 2.94
With glucagon	7.64 ± 4.51
Diagnosis	Number of cases
Isolated GHD	137 (68%)
Hypopituitarism	18 (9%)
Brain tumor	40 (20%)
Syndromes with growth failure	6 (3%)
3 M syndrome	3
Wiedemann–Steiner syndrome	1
Bartter syndrome	2
Patients underwent the ITT and CST (*n* = 119) and were diagnosed with GHD	54 (45.3%)
Patients underwent the GST and CST (*n* = 32) and were diagnosed with GHD	18 (56.2%)
* Total number	201

* The total number represents the patients who were suspected to have GHD and referred to undergo growth hormone provocative tests. BMI = body mass index; CST = clonidine stimulation test; GH = growth hormone; GHD = growth hormone deficiency; GST = glucagon stimulation test; ITT = insulin tolerance test; SD = standard deviation.

**Table 2:** Kappa value of the ITT and CST.

		Value	Asymptotic Standard Error ^a^	Approximate T ^b^	Approximate Significance
Measure of Agreement	Kappa	0.258	0.091	2.816	0.005
N of Valid Cases		119			

^a^ Not assuming the null hypothesis. ^b^ Using the asymptotic standard error assuming the null hypothesis.

**Table 3:** Kappa value of the GST and CST.

		Value	Asymptotic Standard Error ^a^	Approximate T ^b^	Approximate Significance
Measure of Agreement	Kappa	0.178	0.183	1.007	0.314
N of Valid Cases		32			

^a^ Not assuming the null hypothesis. ^b^ Using the asymptotic standard error assuming the null hypothesis.

**Table 4:** (A) Spearman’s rank correlation coefficient of ITT and CST and (B) Spearman’s rank correlation coefficient of the GST and CST.

**A: Spearman’s Rank Correlation Coefficient (ITT and CST)**				
			ITT	CST
Spearman’s rho	ITT	Correlation Coefficient	1.000	0.710
		Significance (2-tailed)		<0.001
		N	119	119
	CST	Correlation Coefficient	0.710	1.000
		Significance (2-tailed)	<0.001	
		N	119	119
**B: Spearman’s Rank Correlation Coefficient (GST and CST)**				
			GST	CST
Spearman’s rho	GST	Correlation Coefficient	1.000	0.702
		Significance (2-tailed)		<0.001
		N	32	32
	CST	Correlation Coefficient	0.702	1.000
		Significance (2-tailed)	<0.001	
		N	32	32

## Data Availability

The data presented in this study are available on request from the corresponding author.
